# Human Cytomegalovirus Infection Changes the Pattern of Surface Markers of Small Extracellular Vesicles Isolated From First Trimester Placental Long-Term Histocultures

**DOI:** 10.3389/fcell.2021.689122

**Published:** 2021-09-10

**Authors:** Mathilde Bergamelli, Hélène Martin, Mélinda Bénard, Jérôme Ausseil, Jean-Michel Mansuy, Ilse Hurbain, Maïlys Mouysset, Marion Groussolles, Géraldine Cartron, Yann Tanguy le Gac, Nathalie Moinard, Elsa Suberbielle, Jacques Izopet, Charlotte Tscherning, Graça Raposo, Daniel Gonzalez-Dunia, Gisela D’Angelo, Cécile E. Malnou

**Affiliations:** ^1^Institut Toulousain des Maladies Infectieuses et Inflammatoires (Infinity), INSERM, CNRS, UPS, Université de Toulouse, Toulouse, France; ^2^Service de Néonatalogie, CHU Toulouse, Hôpital des Enfants, Toulouse, France; ^3^Laboratoire de Biochimie, CHU Toulouse, Hôpital Rangueil, Toulouse, France; ^4^Laboratoire de Virologie, CHU Toulouse, Hôpital Purpan, Toulouse, France; ^5^CNRS UMR 144, Structure et Compartiments Membranaires, Institut Curie, Université Paris Sciences et Lettres, Paris, France; ^6^CNRS UMR 144, Plateforme d’Imagerie Cellulaire et Tissulaire (PICT-IBiSA), Institut Curie, Université Paris Sciences et Lettres, Paris, France; ^7^Service de Diagnostic Prénatal, CHU Toulouse, Hôpital Paule de Viguier, Toulouse, France; ^8^INSERM UMR 1027, UPS, Equipe SPHERE Epidémiologie et Analyses en Santé Publique: Risques, Maladies Chroniques et Handicaps, Université de Toulouse, Toulouse, France; ^9^Service de Gynécologie Obstétrique, CHU Toulouse, Hôpital Paule de Viguier, Toulouse, France; ^10^Développement Embryonnaire, Fertilité, Environnement (DEFE), INSERM UMR 1203, Université de Toulouse et Université de Montpellier, Montpellier, France; ^11^CECOS, Groupe d’Activité de Médecine de la Reproduction, CHU Toulouse, Hôpital Paule de Viguier, Toulouse, France

**Keywords:** early placenta, extracellular vesicles, congenital infection, human cytomegalovirus, placental histoculture

## Abstract

Extracellular vesicles (EVs) have increasingly been recognized as key players in a wide variety of physiological and pathological contexts, including during pregnancy. Notably, EVs appear both as possible biomarkers and as mediators involved in the communication of the placenta with the maternal and fetal sides. A better understanding of the physiological and pathological roles of EVs strongly depends on the development of adequate and reliable study models, specifically at the beginning of pregnancy where many adverse pregnancy outcomes have their origin. In this study, we describe the isolation of small EVs from a histoculture model of first trimester placental explants in normal conditions as well as upon infection by human cytomegalovirus. Using bead-based multiplex cytometry and electron microscopy combined with biochemical approaches, we characterized these small EVs and defined their associated markers and ultrastructure. We observed that infection led to changes in the expression level of several surface markers, without affecting the secretion and integrity of small EVs. Our findings lay the foundation for studying the functional role of EVs during early pregnancy, along with the identification of new predictive biomarkers for the severity and outcome of this congenital infection, which are still sorely lacking.

## Introduction

Long considered as a passive barrier, the placenta is now recognized as a main actor in orchestrating the numerous exchanges between the mother and the fetus, in protecting the fetus against infections and allowing adaptation of the maternal metabolism to pregnancy (McNanley and Woods, 2008; Burton and Fowden, 2015). In the past decade, a new mode of communication of the placenta with both maternal and fetal sides has been described and extensively studied, consisting of the secretion of placental extracellular vesicles (EVs), which increases along the pregnancy and stops after delivery (Luo et al., 2009; Sarker et al., 2014). EVs are membranous nanovesicles released by cells into the extracellular space and body fluids, under physiological and pathophysiological conditions (van Niel et al., 2018; Kalluri and LeBleu, 2020). In a simplistic way, we can distinguish large microvesicles (up to 1 μm), derived from an outward budding of the plasma membrane; and exosomes (ranging from 30 to 200 nm in diameter) generated by inward budding of the membrane of late endosomes, leading to a multivesicular body that will fuse with the plasma membrane and release its content into the extracellular space. Discrimination between the different types of EVs based on their biogenesis pathway and/or physical characteristics is still the subject of many studies and their classification is continuously evolving (Colombo et al., 2014; Kowal et al., 2016; van Niel et al., 2018; Kalluri and LeBleu, 2020). Hence, as it is often difficult to clearly prove the exact nature of exosomes compared to other vesicle subtypes, the term exosome has sometimes been used improperly in the literature, which must be interpreted with caution. Indeed, the literature devoted to placental EVs can sometimes lead to confusion as to the nature of the EVs examined. Some studies have rigorously examined the different categories of EVs, and have underlined the importance of small EVs (sEVs, *i.e.*, exosomes) in several pathologies of pregnancy (Adam et al., 2017; Salomon and Rice, 2017; Sadovsky et al., 2020), even if large EVs (also called microvesicles) also play an undeniable role during pregnancy. Notably, an antiviral role has been specifically attributed to sEVs derived from isolated cytotrophoblasts of term placenta (Delorme-Axford et al., 2013; Ouyang et al., 2016). We have, therefore, chosen to focus on sEVs and to use this terminology, in accordance with the ISEV guidelines (Thery et al., 2018).

Interestingly, placental sEVs are detected in the maternal serum during pregnancy and their composition is altered upon placental pathologies such as diabetes mellitus, intrauterine growth restriction or preeclampsia (Salomon et al., 2016; Cuffe et al., 2017; Chiarello et al., 2018; Miranda et al., 2018; Herrera-Van Oostdam et al., 2019; Kandzija et al., 2019; Malnou et al., 2019). Thus, sEVs may represent valuable non-invasive biomarkers reflecting the status of the placenta and of the pregnancy (Mitchell et al., 2015; Jin and Menon, 2018). Moreover, as sEVs can be internalized by recipient cells and exert biological function, any alteration of their cargo may modify their normal activity (Kalluri and LeBleu, 2020; Sadovsky et al., 2020). In this context, it is important to develop relevant models which allow preparation of placental sEVs in a robust and reproducible manner, in order to guarantee their use for downstream analysis. In this regard, early placentas appear to be well-suited experimental models, since many pregnancy pathologies and developmental defects are the result of placental insults occurring during the first trimester of pregnancy (Montiel et al., 2013; O’Tierney-Ginn and Lash, 2014; Silasi et al., 2015). In this context, the use of tissue explants is particularly relevant since they preserve the tissue cyto-architecture. This allows deciphering the complex mechanisms of (patho)physiological processes, and enables the study of sEV secretion over several days, which is not feasible with most other currently available models (Grivel and Margolis, 2009; Fitzgerald et al., 2018).

Among many environmental agents, viral congenital infections are a major cause of impaired placental and fetal development. Infection by human cytomegalovirus (hCMV) concerns 1% of live births in developed countries and is responsible for various placental and fetal damage, especially at the level of the fetal central nervous system, leading to diverse brain disorders (Kenneson and Cannon, 2007; Cannon et al., 2010; Lanzieri et al., 2015). Currently, non-invasive diagnostic tools to assess fetal hCMV infection are lacking and very few, easily implementable methods to predict fetal impairment exist, especially concerning neurosensorial damage (Benoist et al., 2008; Guerra et al., 2008; Leruez-Ville and Ville, 2017). Thus, the identification of non-invasive diagnosis and prognosis biomarkers within sEVs would be a great step forward in assessing placental and fetal damage and would provide a valuable decision support tool. Finally, to date, the functional role of sEVs in the context of placental infection by hCMV and their contribution to placental dysfunction are not known.

In this work, we have optimized a histoculture model of first trimester placental explants, previously developed in our team (Lopez et al., 2011; Benard et al., 2014), to isolate sEVs with a purity compatible with analyses of their composition and features. This model, permissive to hCMV infection (Lopez et al., 2011; Benard et al., 2014), enabled the purification of sEVs devoid of contaminant viral particles. We showed that the secretion and integrity of sEVs was preserved upon hCMV infection, with significant modifications in the expression levels of sEV surface proteins. Thus, this model opens up promising prospects for modeling chronic stresses at the start of pregnancy, including viral infections, to identify biomarkers necessary to detect very early placental and fetal damage and to study the physiological relevance of sEVs for placental development and function.

## Materials and Methods

### Human Ethic Approval

The biological resource center Germethèque at the Toulouse site (BB-0033-00081) obtained the consent form from each patient for the use of samples included in this study (CPP.2.15.27) in order to carry out the research program. The steering committee gave its approval for the realization of this study on Feb 5^th^, 2019. The hosting request made to Germethèque bears the number 20190201 and its contract is referenced under the number 19 155C. The biological resource center has a declaration DC-2014-2202 and an authorization AC-2015-2350. In accordance with privacy policies concerning voluntary pregnancy termination, the only collected associated clinical data was the term at which the pregnancy was terminated (in weeks) and the age of the women at the time of pregnancy termination.

### hCMV Viral Strain, Viral Stock Production and Titration

The viral strain of hCMV used in this study is the endotheliotropic VHL/E strain (a kind gift from C. Sinzger, University of Ulm, Germany) (Stegmann et al., 2019). Viral stocks were made by amplification of the virus on MRC5 cells and concentration by ultracentrifugation, as described previously (Rolland et al., 2016). Virus titration was determined by indirect immunofluorescence assays against the Immediate Early (IE) antigen of hCMV upon infection of MCR5 by serial dilutions of the viral stock (Rolland et al., 2016). Additionally, virus titration was also performed by qPCR as described on viral stocks and placental histoculture supernatants (Mengelle et al., 2016).

### Placental Histoculture and Infection

**FIGURE 1:**
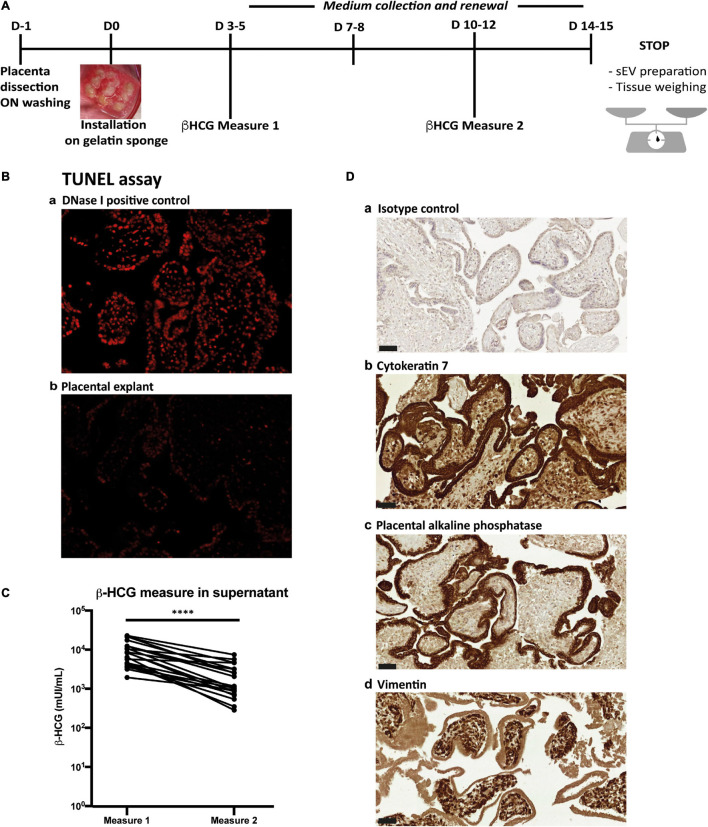
Placental histoculture set-up and characterization. **(A)** Pipeline of placental histoculture model. **(B)** Fluorescence-based TUNEL assay done on cross section of placental villi from histoculture at day 14. a- DNase I positive control; b-TUNEL assay. Image representative from three independent experiments. **(C)** β-HCG measurements in histoculture supernatants. For each placental histoculture, β-HCG measurement was realized in the supernatant between days 3–5 (measure 1) and days 10–12 (measure 2). ^****^ *p* < *0.0001* by paired *t*-test (*n* = 21 independent histocultures). **(D)** Cross sections of immunohistochemistry and hematoxylin staining of placental villi from histoculture at day 14, observed by bright field microscope. a- Isotype control; b- Cytokeratin 7; c- PLAP; d- Vimentin. Image representative from at least three independent experiments. Scale bar = 50 μm.

Placental histocultures were adapted from the model we previously described and validated (Figure 1A) (Rauwel et al., 2010; Lopez et al., 2011; Benard et al., 2014). First trimester placentas [21 placentas; mean = 11.72 ± 0.39 (SEM) weeks of amenorrhea, *i.e.*, 9.72 ± 0.39 weeks of pregnancy; age of the women: mean = 30.71 ± 2.35 (SEM) year-old] were collected following elective abortion by surgical aspiration at Paule de Viguier Maternity Hospital (Toulouse, France) by the medical team, and immediately subjected to dissection. Isolation of trophoblastic villi was performed from total placental tissue by manual dissection in Phosphate Buffer Saline (PBS), with particular care to exclude decidua, membranes and umbilical cord. Tissues were repeatedly washed in PBS to eliminate red blood cells. Each placenta was dissected in small pieces (2–3 mm^3^) and kept overnight in “Exofree” medium (see “Isolation of sEVs” section) in a 5% CO_2_ incubator at 37°C, to eliminate the remaining red blood cells. To infect placental explants by hCMV upon dissection, an overnight incubation was performed with 500 μl of hCMV pure viral stock (corresponding to around 10^8^ focus forming units, ffu) mixed with 500 μl of Exofree medium. The day after (day 0), explants were washed six times in PBS and installed, nine by nine, on re-hydrated gelatin sponges (Gelfoam, Pfizer) in each well of a 6-well plate containing 3 ml of Exofree medium (Figure 1A). A minimum of six wells, *i.e.*, 54 explants, were used per placenta for each experimental condition (*i.e.*, non-infected or infected). Conditioned medium was collected and completely replaced with fresh Exofree medium every 3 to 4 days for the duration of the culture.

To maximize the recovery of sEVs and obtain a rate production compatible with further analyses, conditioned media obtained were pooled until EV purification, for each experimental condition for one given placenta. At two time points during culture, 300 μl of culture supernatants were used to measure β-Human Chorionic Gonadotropin (β-HCG) levels. Free β-HCG was measured on a COBAS system (Roche Diagnostics, Switzerland), modular analytics E170, cobas e601 according to manufacturer protocol (Application Code Number 033) and according to a published method (Sturgeon and McAllister, 1998). In addition to β-HCG dosage, release of virus by infected explants, indicating active viral replication, was assessed by hCMV qPCR titration on supernatant, as described above (Mengelle et al., 2016).

At the end-point of the histoculture, total collected medium was used to perform sEV isolation. Placental explants were weighed in order to normalize, calculate sEV yield and define an appropriate resuspension volume upon sEV preparation. Three explants were used for immuno-histochemistry and the others were frozen at −80°C for further analyses.

### Immuno-Histochemistry

Placental explants were fixed in formalin during 24 h at room temperature and embedded in paraffin. Tissue sections (5 μm) were de-waxed using xylene and alcohol and epitope retrieval was carried out using citrate buffer (pH 6) at 95°C during 20 min. Sections were re-hydrated using TBS 0.01% Tween 20 for 5 min and blocked with 2.5% horse serum for 20 min. Immunostainings were performed with the following antibodies: rabbit anti-Cytokeratin-7 (Genetex; 2 μg/mL), mouse anti-Vimentin (Santa-Cruz; 2 μg/mL) and mouse anti-placental alkaline phosphatase (Biolegend; 1 μg/mL). Immunostaining for hCMV was performed as previously described (Benard et al., 2014), using a mouse monoclonal antibody directed against the hCMV IE antigen (clone CH160, Abcam). Secondary antibody-coupled to biotin was then used prior to Vectastain RTU elite ABC Reagent (Vector laboratories) and staining by diaminobenzidine (DAB). Sections were finally counter-stained with hematoxylin. Image acquisition was performed on a Leica DM4000B microscope or on a Panoramic 250 scanner (3DHISTECH).

### TUNEL Assay

TUNEL assay was done using Click-iT Plus TUNEL Assay for *In Situ* Apoptosis Detection kit (Life Technologies), following manufacturer instructions. Briefly, paraffin-embedded tissue sections were de-waxed using xylene and alcohol and fixed in 4% PFA during 15 min at 37°C. After two washes in PBS, permeabilization was realized with proteinase K during 30 min at 37°C, followed by two other washes and a re-fixation step with 4% PFA during 5 min at 37°C. TUNEL assay was then carried out on tissue sections by following manufacturer protocol, by pretreating one sample by DNAse I during 30 min at 37°C as a positive control. Image acquisition was performed on a Zeiss Axiovert 200 microscope.

### Isolation of sEVs

To purify sEVs from placental histocultures, culture media was depleted beforehand from EVs (Thery et al., 2018). To this aim, Dulbecco’s Modified Eagle Medium (DMEM with Glutamax, Gibco) supplemented with 20% Fetal Bovine Serum (FBS, Sigma-Aldrich) was ultracentrifuged at 100,000 g for 16 h at 4°C (rotor SW32Ti, with maximal acceleration and brake) and filtered at 0.22 μm. “Exofree” medium was then obtained by a 1:1 dilution with DMEM to reach 10% FBS and addition of antibiotics at the following concentrations: 100 U/ml penicillin – 100 μg/ml streptomycin (Gibco), 2,5 μg/ml amphotericin B (Gibco) and 100 μg/ml normocin (Invivogen).

**FIGURE 2:**
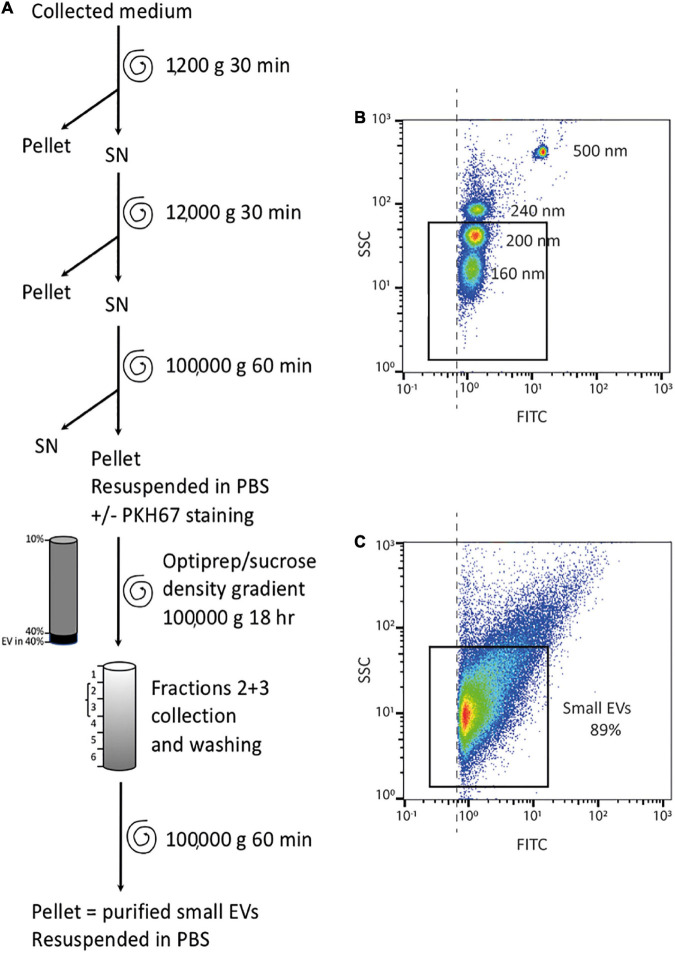
sEV preparation pipeline and flow cytometry analysis. **(A)** Pipeline of sEV preparation using medium collected from placental histocultures. **(B)** Flow cytometry standardization on fluorescent-FITC beads (160 nm, 200 nm, 240 nm, and 500 nm). Each population size was defined on SSC granularity and FITC fluorescence parameters. The black rectangle indicates the gating strategy on small bead populations (160 nm and 200 nm). The dashed line represents the threshold for the detection of sEVs. **(C)** Representative analysis of one placental sEV preparation gated on small events as described above. In this example, 89% of events were below 200 nm.

All steps were then performed at 4°C and PBS solution was filtered on a 0.22 μm filter. Procedures were adapted from Thery et al. (2006), Ouyang et al. (2016), Zicari et al. (2018) according to ISEV guidelines (Thery et al., 2018) and are presented in Figure 2A. From collected histoculture media, several differential centrifugation steps were carried out: a first preclearing centrifugation for 30 min at 1,200 g to eliminate dead cells and large debris, a second ultracentrifugation for 30 min at 12,000 g (rotor SW32Ti, with maximal acceleration and brake) to eliminate large EVs (principally microvesicles), and a last ultracentrifugation of the remaining supernatant for 1 h at 100,000 g (Rotor SW32Ti, with maximal acceleration and brake) allowed to pellet sEVs. The pellet was then resuspended either in 100 μl PBS or in diluent C (Sigma) in order to stain the vesicles by the lipophilic dye PKH67 (Sigma) according to the manufacturer’s instructions (5 min incubation; 1:1,000 dilution). sEVs were then resuspended in a solution of 40% iodixanol in sucrose and the last purification step was carried out by ultracentrifugation on a discontinuous iodixanol/sucrose gradient (10 to 40% iodixanol) with deposition of the sEVs on the bottom of the tube, during 18 h at 100,000 g (rotor SW41Ti, acceleration 5, no brake). The fractions 2 + 3 of the six fractions harvested were then pooled and washed in 25 ml PBS. After a last ultracentrifugation for 1 h at 100,000 g (Rotor SW32Ti, with maximal acceleration and brake), the sEV pellet was resuspended in PBS, in a volume proportional to the weight of tissue (1 μl PBS per 1 mg tissue) and stored at −80°C. We submitted all relevant data of our experiments to the EV-TRACK knowledgebase (EV-TRACK ID: EV200049) and obtained an EV-METRIC score of 100% (Consortium et al., 2017).

### sEV Flow Cytometry

A Macsquant VYB Flow Cytometer (Myltenyi Biotec) was calibrated using Megamix-plus SSC FITC (Biocytex Stago) beads to standardize sEV measurements. Megamix-plus SSC beads of variable diameters (160 nm, 200 nm, 240 nm, and 500 nm) were separated depending on size using SSC side scatter. A gating strategy was defined on 160 nm and 200 nm beads populations to analyze events of size below 200 nm (Figure 2B).

sEV preparations, previously stained with PKH67 as described above, were diluted 1:200 in filtered PBS and analyzed with the same parameters as those used for calibration beads. Gating on events of size below 200 nm allowed count of sEVs and calculation of their concentration for each preparation (Figure 2C). Each sample was analyzed twice. Data were then analyzed with FlowJo software (BD).

### Nanoparticle Tracking Analysis

sEV preparations were diluted 1:100 in filtered PBS (0.2 μm) and tracked using a NanoSight LM10 (Malvern Panalytical) equipped with a 405 nm laser. Videos were recorded three times during 60 s for each sample at constant temperature (22°C) and analyzed with NTA Software 2.0 (Malvern instruments Ltd). Data were analyzed with Excel and GraphPad Prism (v8) softwares.

### Transmission Electron Microscopy and Immunolabeling Electron Microscopy

Procedures were performed essentially as described (Raposo et al., 1996; Hurbain et al., 2017).

For transmission electron microscopy (TEM), sEV preparations were loaded on copper formvar/carbon coated grids (Ted Pella). Fixation was performed with 2% paraformaldehyde in 0.1 M phosphate buffer (pH 7.4), followed by a second fixation with PBS 1% glutaraldehyde in PBS. Samples were stained with 4% uranyl acetate in methylcellulose.

For immunolabeling electron microscopy (IEM), sEV preparations were loaded on grids and fixed with 2% paraformaldehyde in 0.1 M phosphate buffer (pH 7.4). Immunodetection was performed with a mouse anti-human CD63 primary antibody (Abcam ab23792). Secondary incubation was next performed with a rabbit anti mouse Fc fragment (Dako Agilent Z0412). Grids were incubated with Protein A-Gold 10 nm (Cell Microscopy Center, Department of Cell Biology, Utrecht University). A second fixation step with 1% glutaraldehyde in PBS was performed. Grids were stained with uranyl acetate in methylcellulose.

All samples were examined with a Tecnai Spirit electron microscope (FEI, Eindhoven, The Netherlands), and digital acquisitions were made with a numeric 4k CCD camera (Quemesa, Olympus, Münster, Germany). Images were analyzed with iTEM software (EMSIS) and statistical studies were done with Prism-GraphPad Prism software (v8).

### Multiplex Bead-Based Flow Cytometry Assay

sEV preparations were subjected to bead-based multiplex EV analysis by flow cytometry using the MACSPlex Exosome Kit, human (Miltenyi Biotec), according to the manufacturer’s instructions (Koliha et al., 2016).

Briefly, sEV preparations were incubated overnight with 39 different bead populations, each coupled to a different capture antibody. The different bead populations are distinguishable by flow cytometry by a specific PE and FITC labeling. sEVs bound to the beads were then detected with a cocktail composed by anti-CD63, anti-CD9, and anti-CD81 antibodies coupled to APC. Beads coupled to isotype control antibodies were used to assess potential non-specific binding of sEVs. Background was also defined by performing the analysis without any sEVs.

Flow cytometry analysis was performed with a MACSQuant Analyzer 10 flow cytometer (Miltenyi Biotec). The tool MACSQuantify was used to analyze flow cytometry data (v2.11.1746.19438). The background signals were subtracted from the signals obtained for beads incubated with sEVs. GraphPad Prism (v8) software was used to perform statistical analysis of the data.

### Western Blot

sEV samples were lysed in non-reducing conditions in Laemmli buffer, heated for 5 min at 95°C, and loaded on mini protean TGX precast 4-20% gradient gels (Biorad) in Tris-glycine buffer for electrophoresis at 110 V for 2 h. Proteins were electro-transferred onto nitrocellulose membranes using the trans-blot turbo transfer system (Biorad) and membranes were blocked with Odyssey blocking buffer (Li-Cor Biosciences) for 1 h. Membranes were then incubated with primary antibodies: mouse anti-CD81 (200 ng/ml, Santa-Cruz), mouse anti-CD63 (500 ng/ml, BD Pharmingen) or mouse anti-CD9 (100 ng/ml, Millipore) overnight at 4°C in Odyssey blocking buffer, followed by incubation with the secondary antibody IRDye 700 goat anti-mouse IgG (Li-Cor Biosciences), for 1 h at room temperature. Membranes were washed three times in TBS 0.1% Tween 20 during 10 min after each incubation step and visualized using the Odyssey Infrared Imaging System (LI-COR Biosciences).

## Results

To isolate sEVs and standardize their production from placental tissue, we optimized a placental histoculture protocol previously developed and characterized by our team (Lopez et al., 2011; Benard et al., 2014). First trimester placenta explants were cultured and sampled at different time points (Figure 1A). At the end of the culture, tissue sections showed nearly no apoptotic cells as assessed by TUNEL assay (Figure 1B). The secretion of high levels of β-HCG confirmed the viability and secretory capacity of placental explants (Polliotti et al., 1995) (Figure 1C). In agreement with our previous studies (Lopez et al., 2011), β-HCG levels gradually decreased, but remained sustained throughout the experiment. Assessment of tissue architecture and integrity was also performed at the end of the culture; tissue sections were examined for the expression of Cytokeratin-7 (CK-7; trophoblast marker), placental alkaline phosphatase (PLAP; syncitiotrophoblast marker) and Vimentin (mesenchymal cell marker). We observed a typical double layer of trophoblastic cells, consisting of an outer syncitiotrophoblastic layer and an inner cytotrophoblastic layer, surrounding the villous stroma (Figure 1D). These results indicate that the trophoblastic villi architecture is well preserved during the culture, consistent with our previous works (Lopez et al., 2011; Benard et al., 2014).

We next designed a protocol to maximize the recovery of sEVs and allow their detailed characterization (Figure 2A). After the gradient ultracentrifugation step, the majority of sEVs was found in fractions 2 and 3, corresponding to a density of 1.086 and 1.116, respectively, consistent with the density expected for sEVs (Kowal et al., 2016; Ouyang et al., 2016). The last sEV pellet was dissolved into a final volume of PBS proportional to tissue weight, to normalize and compare sEV yields between experiments. Except when preparations were used for multiplex bead-based flow cytometry assays, sEVs were stained with the fluorescent lipophilic dye PKH67 before gradient ultracentrifugation, to allow their counting by flow cytometry (Figure 2A). The flow cytometer was calibrated with FITC-fluorescent beads of different sizes to define gating parameters before analysis of sEV preparations (Figure 2B). Only vesicles smaller than 200 nm of diameter were counted; the majority of the analyzed events displayed an approximate size smaller than 160 nm (Figure 2C). The average yield obtained was of 29,459 ± 5,370 sEV per mg of tissue (mean ± SEM).

**FIGURE 3:**
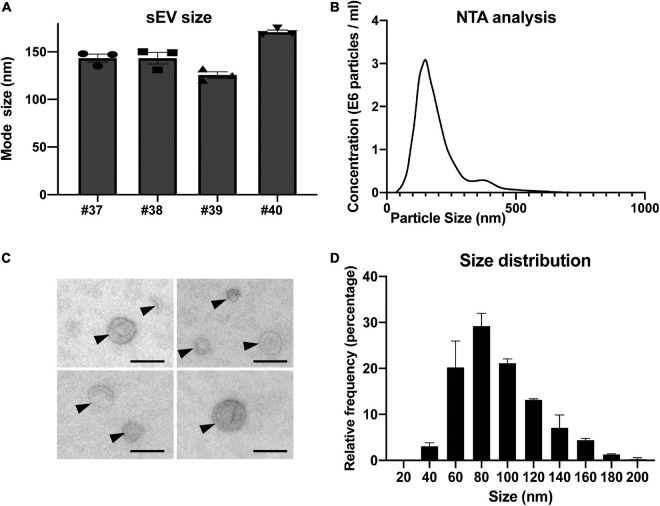
Nanoparticle tracking analysis and electron microscopy characterization of sEV prepared from placental explants. **(A)** Individual analyses of mode size (nm) of four independent placental sEV preparations by nanoparticle tracking analysis. Histograms represent mean ± SEM of three independent measurements (represented by individual dots) for each preparation. **(B)** One representative nanoparticle tracking analysis. **(C)** Placental sEVs observed by TEM of two independent experiments. sEVs are indicated by black arrows. Scale bar = 100 nm. **(D)** Frequency distribution analysis of placental sEV size. Each bar of the histogram represents the mean ± SEM of the relative frequency per bin (bin width = 20 nm) of two independent experiments. Total sEV count was 172 and 208 for each sEV preparation, respectively.

To further describe the population of purified sEVs, we performed nanoparticle tracking analyses (NTA). Concentrations of sEVs determined for four independent preparations laid in the same range as the ones determined by flow cytometry and the comparison of the results obtained by the two methods showed no significant difference (*p* = *0.3415* by paired-*t* test; Supplementary Table 1). The mode sizes for the four sEV preparations determined by NTA ranged between 125.7 and 170.7 nm, with a mean of 145.8 ± 9.3 nm (Figures 3A,B). Next, we performed an exhaustive morphological characterization of placental sEVs by TEM (Figures 3C,D). The preparations were highly enriched in vesicles with the typical membranous appearance of sEVs (Figure 3C and Supplementary Figure 1). The average relative size of sEV was determined using isolated sEVs from two independent preparations. By focusing only on selected sEVs according to their structures, we measured an average diameter of 97 and 91 nm for both preparations. More precisely, by observing sEV size distribution, the majority of sEV diameters was around 80 nm (Figure 3D).

**FIGURE 4:**
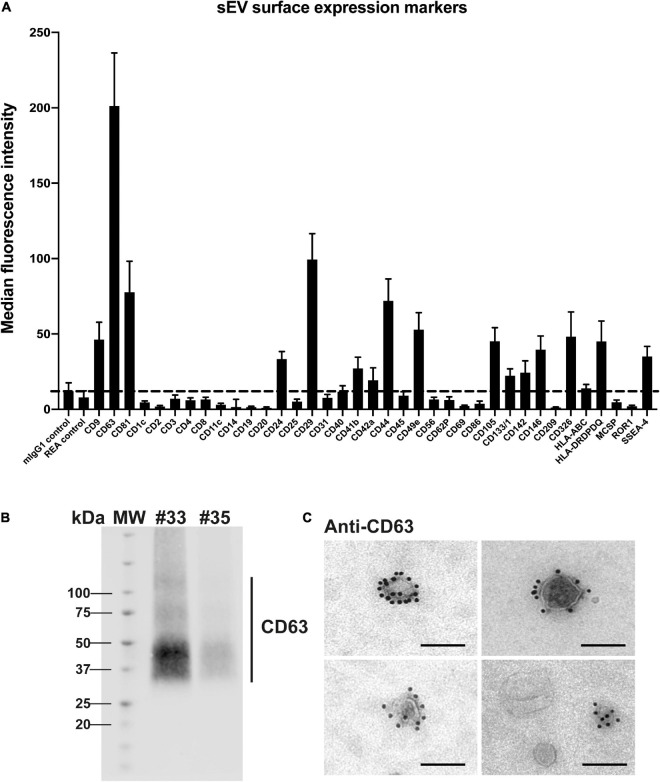
Analysis of placental sEV surface proteins. **(A)** Surface expression level of several proteins of sEVs, based on the multiplex flow cytometry MACSPlex exosome kit assay. Each bar of the histogram represents the mean ± SEM calculated for 6 independent experiments, expressed in Median Fluorescence Intensity for different sEV markers indicated on the X axis. The dashed line represents the detection limit of the test (defined by the two first controls on the left of the histogram). **(B)** Representative western blot analysis against CD63 performed on two independent sEV preparations (#33 and #35). CD63 appears as a smear, since the non-reducing conditions of the western blot preserve its rich glycosylated pattern. MW = molecular weight. **(C)** Placental sEV were immunogold-labeled for CD63, revealed with Protein A-gold particle of 10 nm diameter and observed by TEM. Scale bar = 100 nm (*n* = 2).

Next, a multiplex bead-based flow cytometry assay was carried out to establish a map of sEV surface markers (Figure 4A). An assay enabling the simultaneous detection of up to 37 different EV surface markers in a semi-quantitative way was used (Koliha et al., 2016; Wiklander et al., 2018). We observed a highly positive signal for the canonical tetraspanin CD63, known to be enriched in endosome-derived exosomes, that was also detected by western blot in sEV preparations (Figure 4B). As observed in Figure 4C (and in Supplementary Figure 2), the majority of sEVs were strongly positive for CD63 as evaluated by IEM. A manual counting of CD63-positive sEVs among total sEVs indicated that 60.32 and 61.83% sEVs expressed CD63 for two independent preparations. Finally, two other canonical sEV surface proteins, CD9 and CD81, were expressed in the sEV preparations (Figure 4A). Altogether, these data indicate that isolated sEVs show the typical features of canonical exosomes, regarding ultrastructure, size and the presence of typical exosome markers.

The bead-based flow cytometry assay also revealed that several proteins expressed by trophoblastic cells were present at the surface of sEV, including CD24 (previously described on trophoblastic EVs) (Sammar et al., 2017), CD49e (also known as integrin α5) (Lee et al., 2016), CD105 (Gregory et al., 2014), CD146 (also named MCAM) (Higuchi et al., 2003) or CD236 (also known as EpCAM) (Wong et al., 2019). Conversely, expression of non-trophoblastic markers, including CD4, CD8, CD31, CD45 or HLA-ABC (Blaschitz et al., 2001; Lee et al., 2016) was not detected on sEVs preparations. Of note, reported markers of placental mesenchymal stem cells were also found at the surface of isolated sEV like CD29 (also known as integrin β1), CD44 and SSEA-4 (Salomon et al., 2013; Lv et al., 2014), indicating that these cells may also contribute to sEV secretion in the histocultures.

**FIGURE 5:**
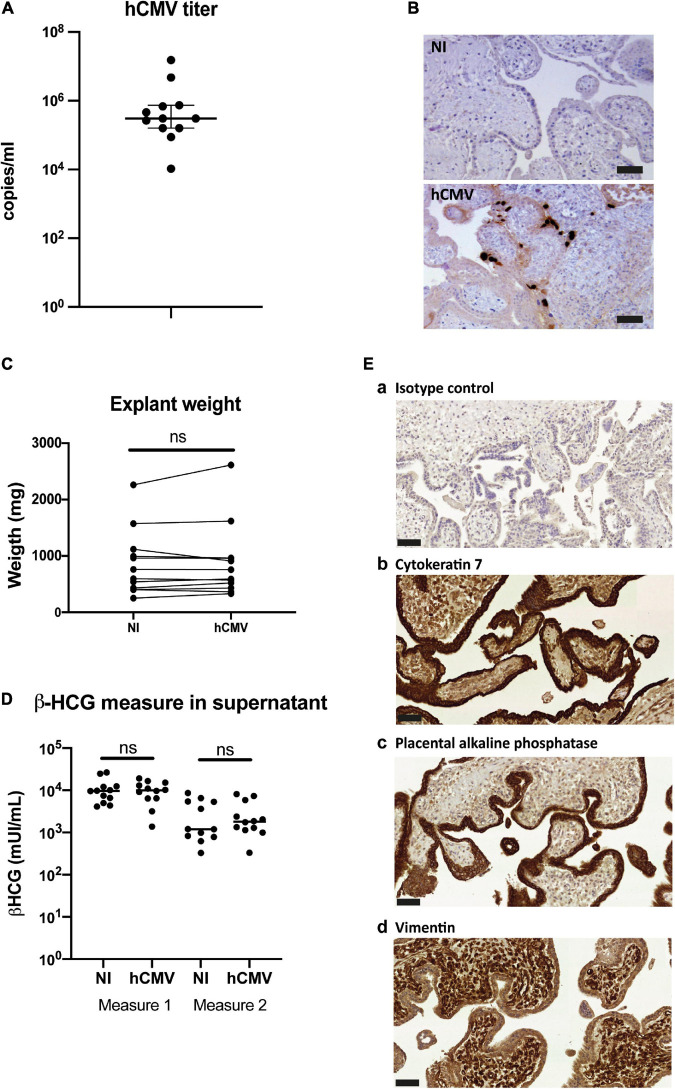
Impact of hCMV infection on placental histocultures. **(A)** Titration of hCMV genome copies released in histoculture supernatant at day 11. On the graph is indicated the median with 95% CI. *n* = 12 independent experiments. **(B)** Representative examples of immuno-histochemistry performed against hCMV IE antigen at day 14 on sections of placental villi, counterstained with hematoxylin. Scale bar = 50 μm. **(C)** Comparison of the explant weight at the end-point of the placental histocultures, performed for twelve independent experiments. Each of the placental explants were pooled per condition and weighted (NI = non-infected). ns, non-significant (*p* = *0.3804*) by Wilcoxon paired test. **(D)** Comparison of the β-HCG secretion in explant supernatants was performed between non-infected (NI) *versus* hCMV-infected placenta, measured with the same timeline as presented in Figure 1A. ns, non-significant (*p* = *0.9697* for measure 1; *p* = *0.5693* for measure 2) by Wilcoxon paired test. *n* = 12 independent experiments. **(E)** Cross sections of immunohistochemistry and hematoxylin staining of hCMV infected placental villi from histoculture at day 14, observed by bright field microscope. a- Isotype control; b- Cytokeratin 7; c- PLAP; d- Vimentin. Image representative from at least three independent experiments. Scale bar = 50 μm.

Next, the impact of hCMV infection on the secretion and characteristics of placental sEVs was examined. Placental explants were infected by the VHL/E clinical strain overnight, extensively washed and maintained in culture during two weeks to favor virus dissemination (Lopez et al., 2011). To monitor virus release into the culture medium, we sampled one aliquot of histoculture supernatant (corresponding to virus released between days 7 and 11), which was analyzed by qPCR. Placental explants displayed active viral release, with hCMV titers in the supernatant comprised between 1.05 × 10^4^ and 1.53 × 10^7^ copies/ml, the median being around 3.04 × 10^5^ copies/ml (Figure 5A). Importantly, these titers were due to virus release and did not correspond to remaining inoculum, since no viral genome could be detected by control qPCR experiments using UV-irradiated virus (data not shown). Moreover, analysis of the tissue sections by immunohistochemistry at the end of the culture confirmed the presence of the IE viral antigen (Figure 5B). Some cells showed intense staining, demonstrating that the virus disseminated well into the tissue after two weeks. Viral infection did not modify the weight of tissue upon culture compared to non-infected conditions (Figure 5C), neither did the levels of secreted β-HCG which remained similar between non-infected and infected placentas for both measures (Figure 5D). Finally, the tissue architecture remained well preserved, as attested by immunohistochemistry performed against CK-7, PLAP, and Vimentin (Figure 5E), thereby ensuring that the sEV preparations were not isolated from dying tissues.

Finally, the characteristics of the sEVs secreted by non-infected or infected placental histocultures were compared. The protocol for sEV preparation, which combines differential ultracentrifugation and gradient ultracentrifugation steps, guaranteed that viral particles did not contaminate sEV preparations, consistent with previous findings (Zicari et al., 2018; Turner et al., 2020). Indeed, hCMV particles are bigger and denser than sEVs and are not co-purified with sEVs upon density gradient ultracentrifugation (Zicari et al., 2018; Turner et al., 2020). The absence of infectious viral particles in sEV fractions was actually confirmed by applying sEVs purified from infected placental explants to MCR5 cells and performing an anti-IE immunofluorescence assay. As expected, no IE expression was detected (Supplementary Figure 3). Moreover, no viral particle was detected by TEM in sEV preparations in all the wide field pictures examined (exemplified in Supplementary Figure 4).

**FIGURE 6:**
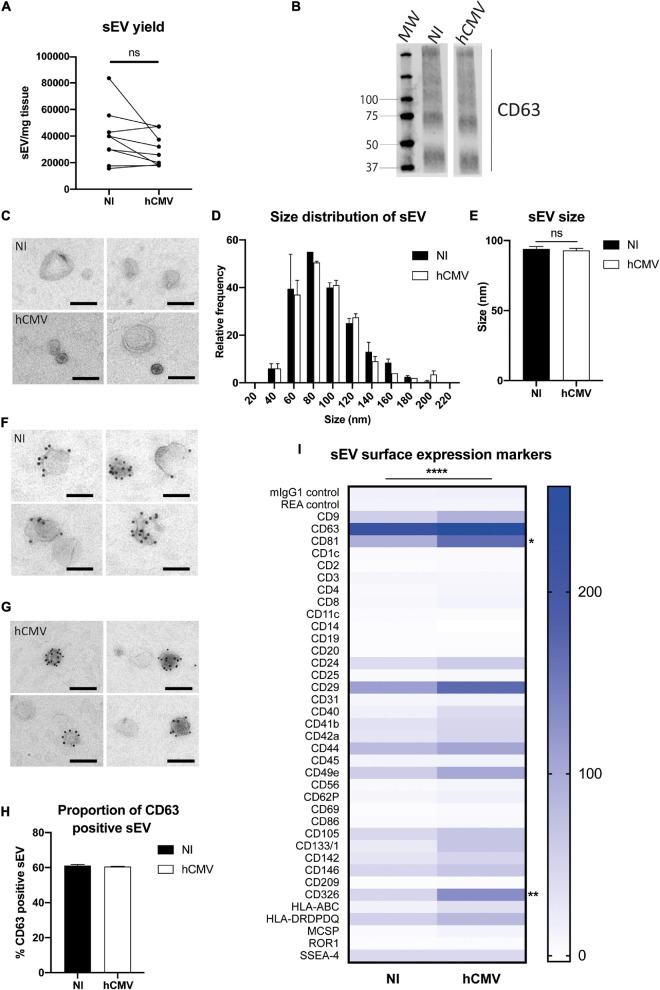
Placental sEV isolation and characterization upon hCMV infection. **(A)** Comparison of the yield of sEV prepared per mg of placental tissue upon sEV preparation between non-infected (NI) *versus* hCMV-infected placental explants. Yield is expressed in sEV number/mg tissue, obtained from nine independent experiments. ns, non-significant by Wilcoxon paired test. **(B)** Western blot analysis against CD63 in sEVs prepared from non-infected (NI) or infected (hCMV) placental explants. This result is representative for at least three independent experiments. CD63 appears as a smear since the non-reducing conditions of the western blot preserves its rich glycosylated pattern. MW = molecular weight. **(C)** Placental sEV isolated from non-infected (NI) *versus* infected (hCMV) placental explants, obtained by TEM. These pictures are representative of two independent experiments. Scale bar = 100 nm. **(D)** Frequency distribution analysis of placental sEV size, compared between non-infected (NI) *versus* infected (hCMV) placental explants. Each bar of the histogram represents the mean ± SEM of the relative frequency per bin (bin width = 20 nm) of two independent experiments. Placental sEV size were measured manually with iTEM measure tool. Total sEV count was 172 and 208 for the NI replicates; 176 and 185 for the hCMV replicates. ns, non-significant (*p* = *0.8814*) by nested *t*-test. **(E)** Mean size ± SEM of placental sEV purified from non-infected (NI) or infected (hCMV) placental explants, calculated from the experiments presented in **(D)**. ns, non-significant (*p* = *0.8871*) by nested *t*-test. **(F,G)** Representative pictures of IEM analysis of placental sEV using antibodies against CD63 (gold bead size = 10 nm), purified from non-infected (NI; F) *versus* infected (hCMV; G) placental explants (*n* = 2). Scale bar = 100 nm. **(H)** Percentage of placental sEV positive for CD63 (at least 1 bead counted per sEV) for two independent experiments. Total sEV count was 189 and 263 for sEV isolated from non-infected placental explants; 143 and 594 for sEV isolated from infected explants. ns, non-significant by nested *t*-test. **(I)** Surface expression level of different proteins of sEV isolated from non-infected (NI) *versus* infected (hCMV) placental explants, based on the multiplex flow cytometry MACSPlex exosome kit assay. Results are represented by a heat-map, calculated from 3 independent experiments for different sEV markers indicated on the left column. Blue intensity is proportional to the level expression calculated in Median Fluorescence Intensity, indicated on the right of the heat-map. ****, p < *0.0001* by 2-way ANOVA for “Infection” factor. Bonferroni *post hoc* comparison test indicated significant increase for CD81 (*, p= *0.0223*) and CD326 (**, p = *0.0029*) for hCMV compared to non-infected (NI) conditions.

When comparing yields of purified sEVs in non-infected *versus* infected histocultures, no significant differences were observed (Figure 6A), indicating that hCMV infection did not affect the global production of sEV by placental tissue. By TEM, sEVs secreted from infected explants displayed the same morphology (Figure 6C and Supplementary Figure 4) and relative size distribution than sEVs isolated from non-infected histocultures (Figure 6D), with no significant difference in their mean size (Figure 6E). sEVs from hCMV-infected explants also expressed CD63, detected both by western blot (Figure 6B) and by IEM (Figures 6F,G and Supplementary Figure 5), being expressed on nearly 60% of the vesicles (Figure 6H).

Finally, the surface expression levels of several proteins expressed by sEVs were examined by multiplex bead-based flow cytometry assay in both conditions (Figure 6I). To perform this assay, quantification of the sEVs by PKH67-based flow cytometry could not be realized, since it would interfere with the assay. Instead, sEV quantity was normalized between non-infected and infected conditions based on the weight of the explants, since hCMV infection did not modify the yield of sEV secretion (Figure 6A). sEVs isolated from hCMV infected explants expressed the same markers than sEVs isolated from non-infected explants, albeit with significant differences for some of them in their expression levels upon infection (Figure 6I). A 2-way ANOVA statistical test confirmed that the infection modified the global pattern of expression of sEV surface proteins (*p* < *0.0001* for the “Infection” factor, no interaction with “Marker” factor). Most of the surface markers expressed in sEV isolated from infected explants showed an increased expression upon infection. Bonferroni’s multiple comparison test indicated that two markers were significantly increased: CD81 (*p* = *0.0223*) and CD326 (EpCAM; *p* = *0.0029*). In conclusion, these findings indicate that hCMV infection of placental explants preserves the secretion of sEVs that conserve the typical characteristics of exosomes with a global modification of the expression levels of some surface proteins.

## Discussion

An increasing number of works focus on placental EVs and their role in physiological and pathological pregnancies (Salomon et al., 2016; Cuffe et al., 2017; Chiarello et al., 2018; Miranda et al., 2018; Herrera-Van Oostdam et al., 2019; Kandzija et al., 2019; Malnou et al., 2019). For these studies, many different models have been used as a source of EVs, both *in vivo*, *ex vivo* or *in vitro*. *In vivo* study of placental EVs isolated from blood is hard to interpret because they come from multiple tissues. The use of placental primary cells or cell lines *in vitro* is very informative, but may lack important aspects of (patho)physiology occurring in a complex tissue architecture, especially during viral infection. An interesting alternative is to use *ex vivo* models to isolate EVs. For term placenta, some studies use dual lobe perfusion performed during 3h to collect EVs (Dragovic et al., 2015; Kandzija et al., 2019), but this system cannot be applied for first trimester placenta and in the context of viral infections that take time to establish in the tissues. Explant culture models, enabling the maintenance of the tissue in culture for several days, have widely been used for different tissues (Grivel and Margolis, 2009), including placenta (Faye et al., 2005; Hamilton et al., 2012; Fitzgerald et al., 2018). Here, we adapted a previously established model of first trimester placental explants, which can be maintained in culture at the air/liquid interface for at least two weeks and is permissive for hCMV replication (Lopez et al., 2011; Benard et al., 2014). Indeed, we could confirm that the integrity of our placental explants was well preserved, consistent with our previous results (Lopez et al., 2011; Benard et al., 2014), with an expected pattern of β-HCG secretion over time (Polliotti et al., 1995) and without overt apoptosis. Moreover, immunohistochemical analyses further established that the complex cytoarchitecture of the trophoblastic villi was also well preserved at the end of the culture, even upon hCMV infection.

From these placental explants, we developed robust and reproducible conditions for the recovery and isolation of sEVs [EV-METRIC score of 100% (Consortium et al., 2017)], in strict accordance with MISEV guidelines (Thery et al., 2018). We unambiguously demonstrated that the sEV preparations were pure and devoid of contaminants, as evidenced by the assessment of multiple parameters. Notably, we showed that sEV preparations presented many features of endosomal-derived exosomes, including membranous vesicles as observed by TEM, an average relative diameter around 95 nm and the presence of exosome components including CD63, CD9, and CD81.

Contrasting with studies where sEVs are isolated from a single cell type, our goal here was to examine the global population of sEVs secreted from trophoblastic villi. Even if the cellular origin of the vesicles is varied, we reasoned that it may better reflect those of the placental environment and better suited to assess the overall changes of the vesicles following stress. As the tissue architecture was well preserved, it is likely that the outer layers of cyto- and syncitiotrophoblasts contributed to sEV secretion. Indeed, proteins expressed by trophoblasts were detected on the sEV surface, including CD326, CD24 or CD49e (Lee et al., 2016; Sammar et al., 2017; Wong et al., 2019). We also observed the presence of proteins described for mesenchymal stem cells, like CD29, CD44 and SSEA-4, suggesting that such cells might contribute to sEV secretion (Lv et al., 2014; Salomon et al., 2014). Of note, although sEV have different cellular origins, the pattern of expression of surface markers was very reproducible among sEV preparations.

To assess whether this model was relevant for evaluating the consequences of a chronic environmental stress on placental sEVs, we sought to examine the impact of hCMV infection on sEVs secreted by the placental villi. We reasoned that analysis of sEVs from first trimester placenta may be particularly relevant considering the pathophysiology of hCMV congenital infections. Indeed, hCMV efficiently disseminates from the mother to the fetus *via* an active replication in the placenta tissue (Pereira et al., 2005, 2017). Consistent with previous works (Amirhessami-Aghili et al., 1987; Lopez et al., 2011; Hamilton et al., 2012; Benard et al., 2014), hCMV disseminated well in the placental explants and was released into the medium. Based on immunohistochemistry data, tissue infection levels were similar to what has been observed on placentas during natural infection (Uenaka et al., 2019). Under our conditions of infection, the placental explants kept the same weight and histological structure, and continued to secrete sEVs at yields comparable to the uninfected explants.

To maximize the recovery of sEVs for a deep characterization and downstream analyses, the histoculture supernatants were pooled along the culture. Although this may either hide fluctuations and/or attenuate transient or late trends induced by infection, we observed significant global changes in the signature of sEV surface markers upon infection by multiplex bead-based cytometry assay. A significant surface expression increase was observed notably for two proteins: CD326 and CD81. Of note, CD326 (EpCAM) has been suggested to play a role in placental development (Nagao et al., 2009). This surface protein is expressed during the first trimester of pregnancy by a subpopulation of actively dividing trophoblast progenitor cells (Wong et al., 2019). Interestingly, trophoblast progenitor cells are targeted by hCMV infection with important consequences on their ability to differentiate and allow placental development (Tabata et al., 2015). In addition, CD81 has been recently described to play a role in hCMV entry (Viswanathan et al., 2017; Fast et al., 2018). In particular, the presence of CD81 at the surface of host cells, along with CD9 and CD44 for which we also detect a trend for increased expression, is thought to be used as a platform for hCMV entry into cells (Viswanathan et al., 2017). Hence, it is tempting to speculate that the secretion of sEVs with this specific modified pattern of surface proteins upon hCMV infection may have a functional role, by influencing viral dissemination into the tissue and/or contributing to placental development defects.

Currently, there is a growing interest for the discovery of biomarkers reflecting both placental and fetal state, within the placental sEVs, and to address the question of their biological relevance in pathophysiology (Mitchell et al., 2015; Cuffe et al., 2017; Jin and Menon, 2018; Malnou et al., 2019). However, in most models of placental explants described to date, sEVs are generally prepared within the first 16 to 48 h of culture (Tong et al., 2016; Tong and Chamley, 2018), a duration that does not allow to evaluate the long-term effects of chronic stress on sEVs. Hence, our model of early placental explants that can be cultured over several days appears as a very valuable tool to evaluate the impact of chronic environmental stress, including viral infection but also hypoxia or endocrine disruptors, on the phenotype and functional role of sEVs secreted by the placenta in early pregnancy. Ultimately, it could also open new perspectives in the search for biomarkers.

## Data Availability Statement

The raw data supporting the conclusions of this article will be made available by the authors, without undue reservation.

## Ethics Statement

The studies involving human participants were reviewed and approved by CPP.2.15.27. The patients/participants provided their written informed consent to participate in this study.

## Author Contributions

MBer, HM, and MM designed and performed the experiments and analyzed data. MBén, MG, GC, YT, and CT collected clinical samples and data. J-MM, JI, and JA performed viral titrations and β-HCG measures. NM from Germethèque was in charge of ethical issues and received women authorizations. IH, GD’A, and GR supervised electron microscopy experiments and data analysis. CM, MM, and CT got grants for this study. CM conceived and designed the study, analyzed data, and wrote the manuscript, with the help of MBer, GD’A, DG-D, ES, and CT. All authors provided critical feedback and helped shape the research, analysis, and manuscript.

## Conflict of Interest

The authors declare that the research was conducted in the absence of any commercial or financial relationships that could be construed as a potential conflict of interest.

## Publisher’s Note

All claims expressed in this article are solely those of the authors and do not necessarily represent those of their affiliated organizations, or those of the publisher, the editors and the reviewers. Any product that may be evaluated in this article, or claim that may be made by its manufacturer, is not guaranteed or endorsed by the publisher.
