# Supplementary material for: Human Cytomegalovirus Infection Changes the Pattern of Surface Markers of Small Extracellular Vesicles Isolated From First Trimester Placental Long-Term Histocultures
Source: Front Cell Dev Biol. 2021 Sep 10;9:689122. doi: 10.3389/fcell.2021.689122 (PMC8461063; doi:10.3389/fcell.2021.689122)

Electron microscopy of small EVs isolated from non-infected placental histoculture
15000x

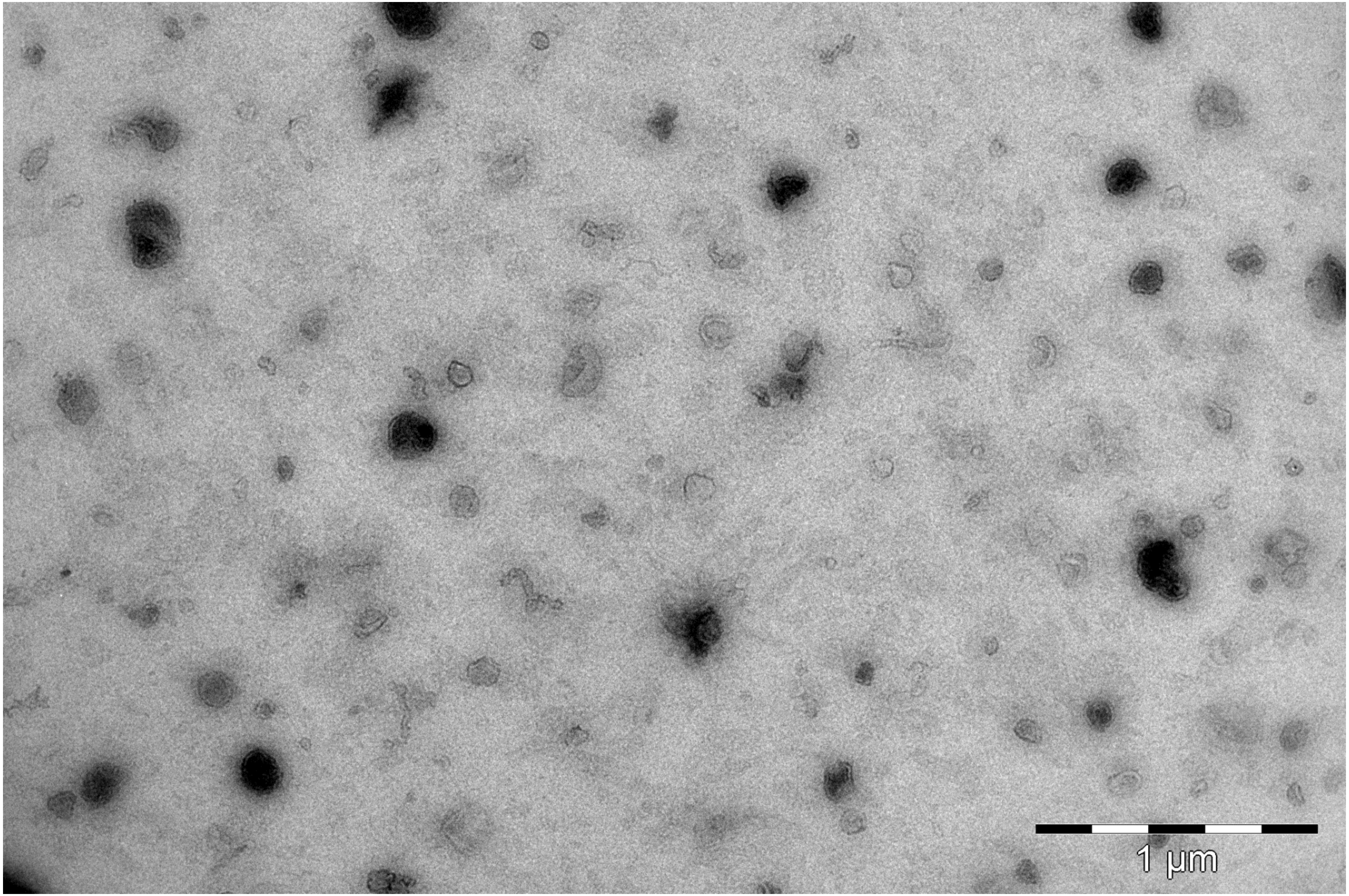

Immuno electron microscopy anti-CD63 of small EVs isolated from non-infected placental histoculture

15000x

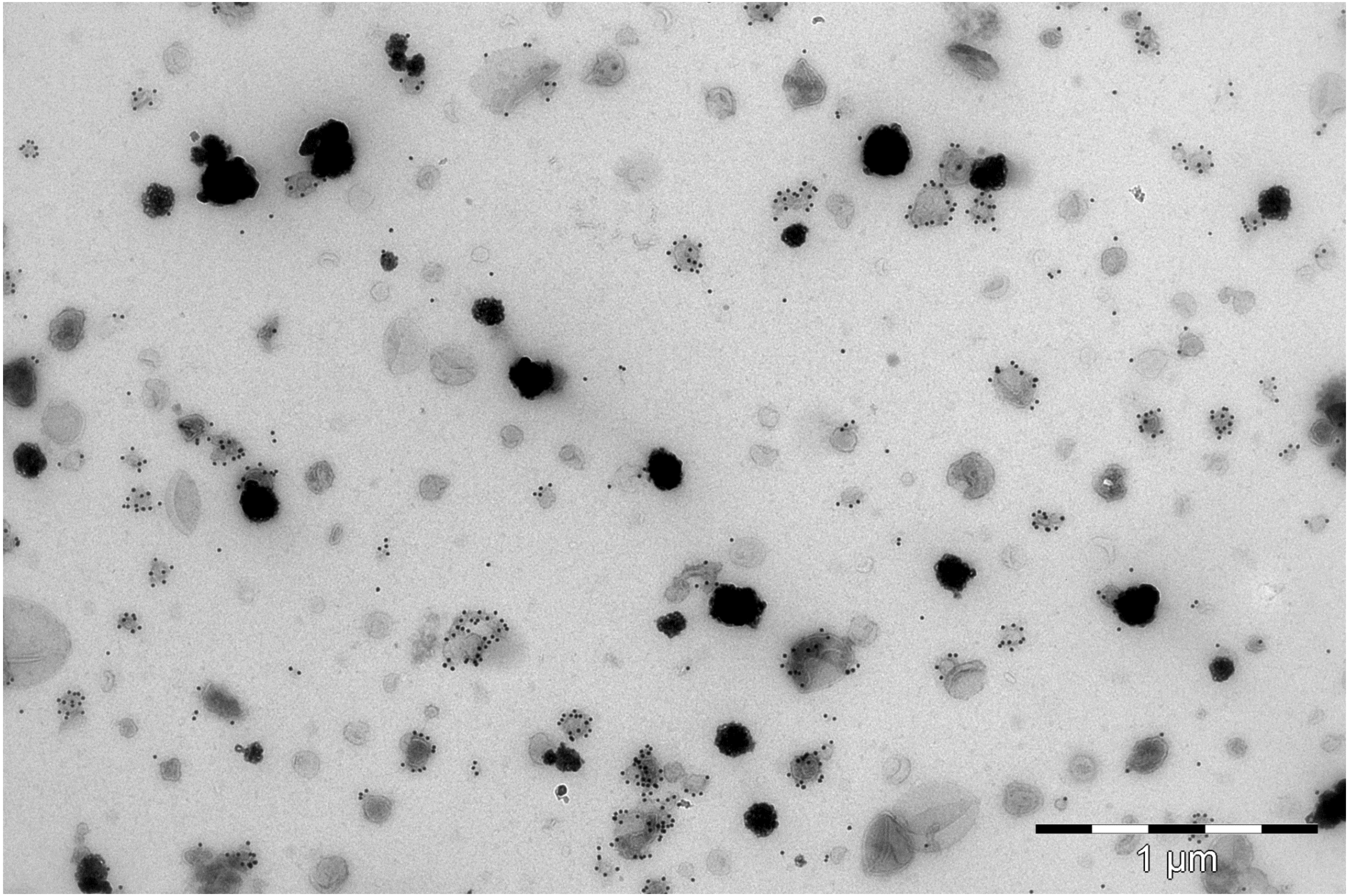

Placental small EVs preparation are devoid of infectious virus. Small EV preparations (left panel) or hCMV at a multiplicity of infection of 10 (right panel) were incubated with MCR5 cells during 24 h. Immunofluorescence was then performed against viral IE antigen (green: IE; blue: DAPI). Magnification = 20 X. The low density of the cells on the right panel is due to the high mortality rate consequent to the virus infection.

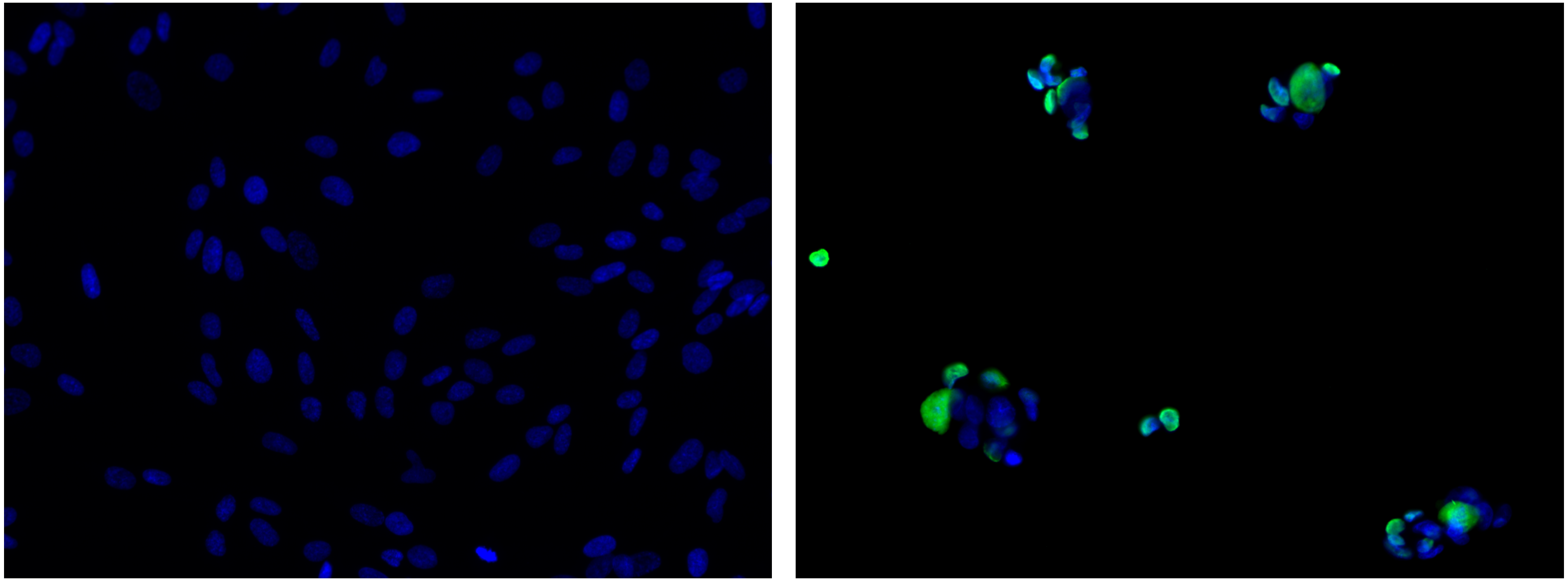

Electron microscopy of small EVs isolated from hCMV-infected placental histoculture
15000x

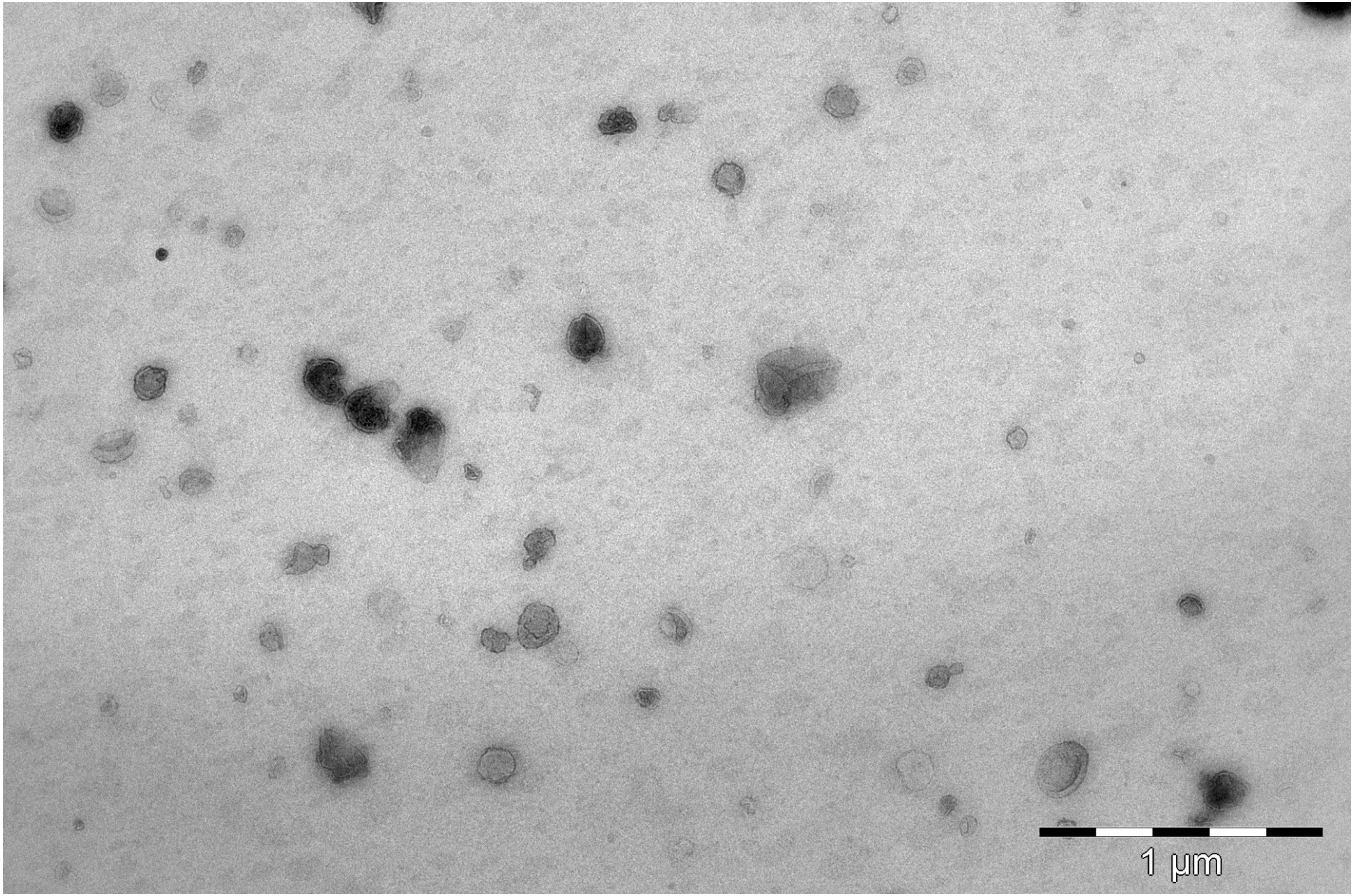

Immuno electron microscopy anti-CD63 of small EVs isolated from hCMV-infected placental histoculture
15000x

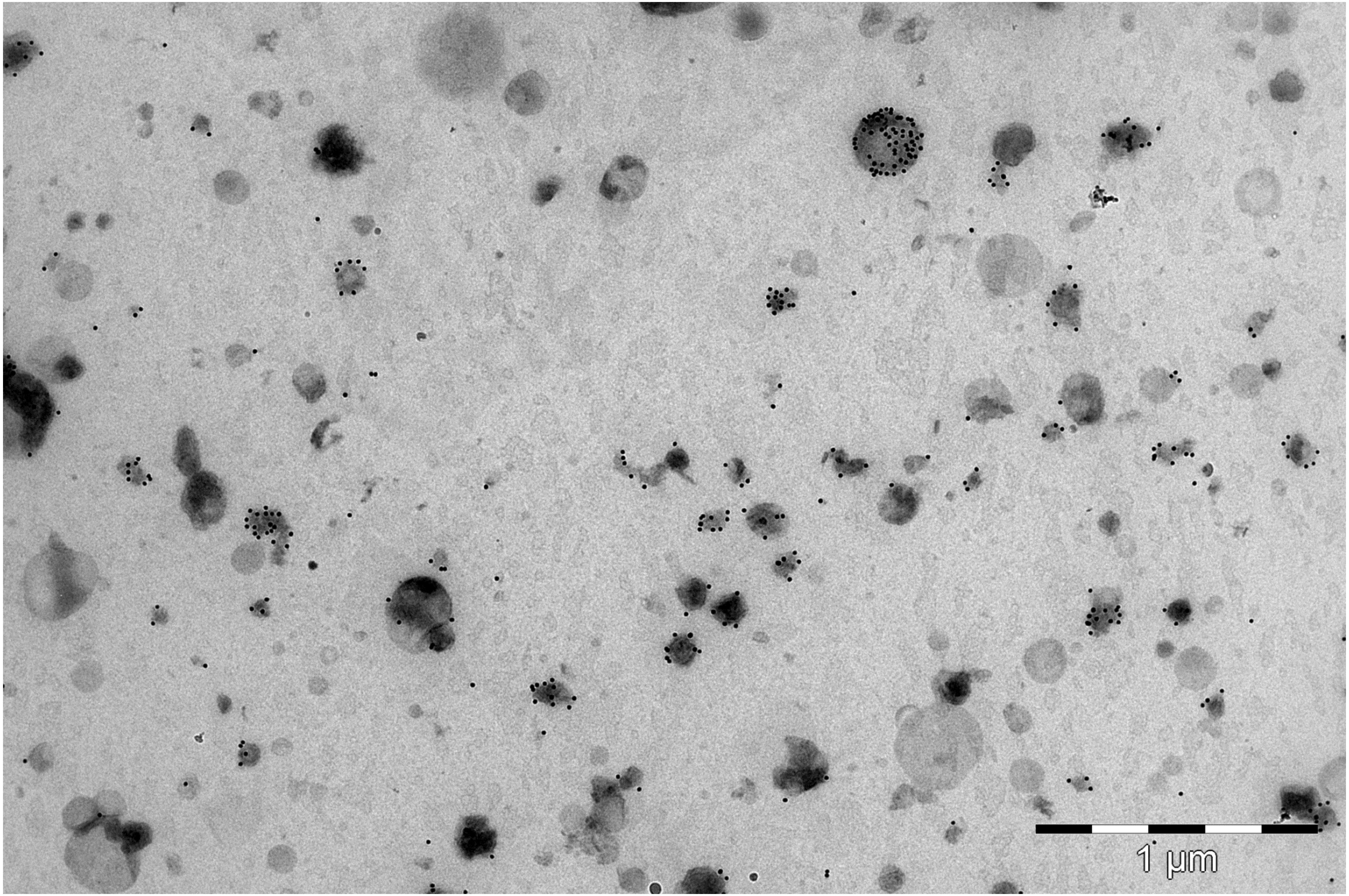

Supplement: Supplementary file 1 [file Presentation_1.pdf]
