# Supplementary material for: Human Cytomegalovirus Infection Changes the Pattern of Surface Markers of Small Extracellular Vesicles Isolated From First Trimester Placental Long-Term Histocultures
Source: Front Cell Dev Biol. 2021 Sep 10;9:689122. doi: 10.3389/fcell.2021.689122 (PMC8461063; doi:10.3389/fcell.2021.689122)
Supplement: Supplementary file 2 [file Table_1.PDF]

Supplementary Table 1: Comparison of sEV counting by flow cytometry and NTA methods

Experiment n°	flow cytometry	NTA
#37	1,45E+08	1,55E+08
#38	8,80E+07	4,08E+08
#39	1,00E+08	1,66E+08
#40	3,00E+08	2,63E+08

Paired-*t* test between flow cytometry and NTA measures
for each experiment shows no significant difference (*p=0,3415*).
